# Short-Chain Fatty Acids in Sepsis: Mechanisms of Action and Therapeutic Advances

**DOI:** 10.3390/biomedicines14091992

**Published:** 2026-09-04

**Authors:** Zhigang Wang, Xiaoyue Wen, Shiying Yuan, Jiancheng Zhang, Dan Xu

**Affiliations:** 1 Department of Critical Care Medicine, Union Hospital, Tongji Medical College, Huazhong University of Science and Technology, Wuhan 430022, China; 18205519798@163.com (Z.W.); 18727272517@sina.cn (X.W.); 2 Key Laboratory of Anesthesiology and Resuscitation, Huazhong University of Science and Technology, Ministry of Education, Wuhan 430048, China; 3 Hubei JiangXia Laboratory, Wuhan 430200, China

**Keywords:** short-chain fatty acids, sepsis, gut microbiota, intestinal barrier, immunometabolism

## Abstract

Sepsis is defined as life-threatening organ dysfunction caused by a dysregulated host response to infection. Its development and progression involve multiple interconnected mechanisms, including uncontrolled inflammation, immunosuppression, metabolic reprogramming, intestinal barrier disruption, and multi-organ injury. Short-chain fatty acids (SCFAs), primarily acetate, propionate, and butyrate, are important metabolites produced by the anaerobic fermentation of dietary fiber and indigestible carbohydrates by gut microbiota. During sepsis, antibiotic exposure, intestinal hypoperfusion, insufficient nutritional substrates, and microbial dysbiosis may deplete SCFA-producing bacteria and lower SCFA levels, thereby aggravating intestinal barrier dysfunction, endotoxin translocation, and systemic inflammatory responses. SCFAs can influence sepsis-associated intestinal, pulmonary, cardiac, hepatic, renal, and cerebral injury by activating receptors such as free fatty acid receptor 2 (FFAR2)/G protein-coupled receptor 43 (GPR43), free fatty acid receptor 3 (FFAR3)/G protein-coupled receptor 41 (GPR41), and G protein-coupled receptor 109A (GPR109A); inhibiting histone deacetylases; and regulating immune-cell metabolism, inflammasome activation, oxidative stress, mitochondrial function, and modes of cell death. In recent years, strategies such as direct SCFA supplementation, promotion of endogenous SCFA production, restoration of SCFA-producing microbial communities, and targeting of SCFA receptors and downstream signaling pathways have shown therapeutic potential. However, their clinical translation remains limited by uncertainties regarding dose, timing, route of administration, patient stratification, and safety. This review systematically summarizes the mechanisms of action and therapeutic advances of SCFAs in sepsis, aiming to provide a reference for microbiome-based interventions and metabolism-targeted therapies in sepsis.

## 1. Introduction

Sepsis is defined as life-threatening organ dysfunction caused by a dysregulated host response to infection and remains a major clinical challenge in critical care medicine. The Global Burden of Disease Study has shown that sepsis continues to impose a substantial global burden, with high numbers of cases and deaths and marked variations across regions, age groups, and healthcare resource settings [1]. Early epidemiological studies also indicated that sepsis is characterized by high incidence, substantial healthcare resource utilization, and high mortality among hospitalized patients [2]. With the introduction of the Sepsis-3 clinical criteria, sepsis-related organ failure assessment (SOFA)-based assessment of organ dysfunction and infection-related risk identification in the context of infection have further emphasized the central role of “dysregulated host response”, rather than infection alone, in the diagnosis of sepsis [3,4].

In recent years, transcriptomic and multi-omics studies have further revealed substantial biological heterogeneity among patients with sepsis. Prospective cohort studies have identified distinct peripheral blood transcriptomic host-response patterns in patients with sepsis, which are associated with clinical outcomes [5]. Other studies have stratified patients with sepsis into different molecular endotypes based on blood gene-expression profiles, suggesting that distinct immunometabolic states may exist under the same clinical diagnosis [6]. Cross-cohort transcriptomic analyses also support the concept that sepsis is not a single disease entity, but rather comprises multiple reproducibly identifiable immune subtypes [7]. Prospective validation of biomarker-based risk models in pediatric sepsis further indicates that host biomarkers may help identify patient populations with different risk profiles [8]. Collectively, these findings suggest that future adjunctive therapies for sepsis should shift from a strategy of generalized anti-inflammatory intervention toward precision approaches guided by host responses, metabolic status, and microbiome-related characteristics.

Against this background, the gut and its microbial ecosystem have become an important focus for sepsis research. Early clinical studies showed that patients with systemic inflammatory response syndrome and critical illness often exhibit marked alterations in the gut microbiota, and that these changes are associated with infectious complications and mortality [9]. Subsequent studies have repeatedly confirmed persistent reductions in fecal SCFAs, decreased microbial diversity, and expansion of opportunistic pathogens in critically ill patients [10,11,12]. Because the gut is involved in nutrient absorption, barrier defense, immune homeostasis, and microbial metabolism, it may serve not only as a target organ of injury in sepsis, but also as an amplifier of systemic inflammation and multiple organ damage.

Short-chain fatty acids (SCFAs) are monocarboxylic acids with short aliphatic carbon chains. In the human gut, SCFAs are produced predominantly through the anaerobic microbial fermentation of dietary fiber, resistant starch, and other fermentable carbohydrates that escape digestion and absorption in the small intestine, although microbial metabolism of proteins and amino acids can also make a minor contribution. The predominant gut-derived SCFAs are acetate (C2), propionate (C3), and butyrate (C4), which together account for the vast majority of SCFAs in the colon. In this review, the term “SCFAs” refers specifically to these three major gut microbiota-derived metabolites unless otherwise stated. In contrast to many pro-inflammatory gut-derived molecules, SCFAs are generally closely associated with the maintenance of intestinal barrier integrity, immune homeostasis, and anti-inflammatory regulation. Human and animal studies have shown that dietary patterns, gut microbiota composition, and the abundance of SCFA-producing bacteria can markedly influence host metabolic and immune status. Importantly, this relationship is highly dynamic. Restriction of fermentable carbohydrate intake can reduce fecal SCFA concentrations and the abundance of SCFA-producing bacteria, whereas both long-term and short-term dietary patterns can reshape microbial community structure and metabolic output [13,14,15]. Because SCFAs can serve as energy substrates for epithelial cells and regulate host signaling through G protein-coupled receptors and epigenetic mechanisms, diet-induced changes in SCFA availability may further influence intestinal barrier function and immune-cell homeostasis. This diet–microbiota–SCFA relationship may be particularly relevant during sepsis, when fasting, low-fiber nutritional intake, and other intensive care unit (ICU)-related interventions may further limit the availability of fermentable substrates. Therefore, SCFAs are not only metabolic products reflecting functional alterations in the gut microbiota, but also important mediators linking the intestinal microecosystem to host inflammatory and immune responses. At present, direct SCFA supplementation, promotion of endogenous SCFA production, restoration of SCFA-producing microbial communities, and modulation of SCFA receptors and their downstream pathways are all considered to have potential as adjunctive therapeutic strategies for sepsis. Animal experiments and some clinical studies have suggested that fecal microbiota transplantation (FMT), SCFA supplementation, probiotics or synbiotics, enteral nutrition, and microbiome-targeted interventions may influence sepsis outcomes through different mechanisms [16,17,18]. In addition, experimental studies have shown that SCFA administration can attenuate neuroinflammation and cognitive dysfunction in sepsis-associated encephalopathy, including through GPR43-dependent effects. Sodium butyrate has also been shown to ameliorate sepsis-associated lung injury by enhancing intestinal and pulmonary barrier function and modulating CD4^+^Foxp3^+^ regulatory T cells. Collectively, these findings further support the notion that SCFAs can influence outcomes in sepsis [19,20,21]. However, the effects of SCFAs are influenced by dose, route of administration, disease stage, microbial background, and the patient’s immune status. Therefore, their mechanistic basis and translational limitations still require systematic evaluation. Accordingly, this review summarizes the physiological basis of SCFAs, their metabolic imbalance in sepsis, mechanisms of action, associations with multiple-organ injury, and therapeutic advances, with the aim of providing a reference for microbiome-based and metabolism-targeted therapies in sepsis.

## 2. Physiological Roles of SCFAs and Their Dysregulated Metabolism in Sepsis

### 2.1. Sources and Types of SCFAs

SCFAs are organic fatty acids with relatively short carbon chains, of which acetate, propionate, and butyrate are the predominant forms in the human gut. SCFAs can cross the colonic epithelium through both nonionic diffusion and carrier-mediated transport. In their anionic form, SCFAs are taken up primarily through transport systems including the H^+^-coupled monocarboxylate transporter 1 and Na^+^-coupled monocarboxylate transporters, although other related transport systems may also contribute. Following uptake by epithelial cells, a substantial proportion of butyrate is locally oxidized and utilized by colonocytes, whereas the remaining SCFAs enter the portal circulation and undergo varying degrees of hepatic first-pass uptake before reaching the systemic circulation. Classical human studies have shown that SCFAs can be detected in the colonic lumen, portal vein, hepatic vein, and peripheral venous circulation, suggesting that they can enter the host circulation from the intestinal lumen and affect distant organs [22]. Intestinal anaerobes generate acetate, propionate, and butyrate through distinct and partially overlapping metabolic pathways, including cross-feeding reactions among microbial taxa. Among these SCFAs, acetate is generally the most abundant, whereas butyrate is preferentially utilized as an energy substrate by colonocytes [22].

The composition of SCFA-producing microbial communities is markedly influenced by diet, antibiotic exposure, host disease status, and intestinal ecological niches. Low-carbohydrate diets can reduce fecal butyrate levels and the abundance of butyrate-producing bacteria, indicating that the supply of dietary substrate is a key determinant of SCFA production [13]. Studies of the human gut microbiota have shown that distinct dominant microbial community structures are closely associated with dietary patterns and metabolic capacity, with diets rich in plant-derived dietary fiber generally favoring the maintenance of a complex and diverse fermentative microbiota [14,23,24]. Short-term dietary changes can also rapidly reshape the gut microbiota and its metabolic output [15], suggesting that SCFA production is highly plastic.

The main types, sources and physiological characteristics of SCFAs are provided in Table 1.

### 2.2. Absorption, Transport, and Signal Recognition of SCFAs

SCFAs are not only energy substrates but also important signaling molecules. Acetate and propionate more readily enter the portal and peripheral circulation, whereas butyrate plays a central role in local metabolism within colonic epithelial cells. Importantly, SCFAs detected in the circulation should not be assumed to originate exclusively from gut microbial fermentation. Their peripheral concentrations reflect the combined effects of intestinal microbial production and absorption, first-pass hepatic metabolism, tissue utilization, endogenous host metabolism, and clearance. This distinction is particularly relevant for acetate, which can also be generated through host metabolic pathways. Therefore, circulating SCFA concentrations should not be interpreted as a direct surrogate for bacterial SCFA production, especially in the metabolically disturbed state of sepsis. Animal and cellular studies have shown that butyrate promotes the assembly of intestinal epithelial tight junctions and improves barrier function, an effect associated with energy-sensing pathways such as AMP-activated protein kinase (AMPK) [27]. In addition, gut microbiota-derived SCFAs can enhance intestinal barrier function by stabilizing epithelial hypoxia-inducible factor-related pathways, indicating that their effects extend beyond energy provision to include regulation of epithelial stress adaptation [28].

SCFA recognition is mainly mediated by G protein-coupled receptors, including FFAR2/GPR43, FFAR3/GPR41, and GPR109A. Early pharmacological studies demonstrated that GPR41 and GPR43 can be activated by propionate and other short-chain carboxylic acids [29]. Subsequent studies of human receptor function further confirmed that GPR41 and GPR43 are involved in the sensing and activation of polymorphonuclear leukocytes in response to SCFAs [30]. In addition, GPR41 can mediate the regulatory effects of SCFAs on the sympathetic nervous system and energy homeostasis, suggesting that SCFA signaling may extend beyond the gut to neuroendocrine networks [31].

In addition to receptor-dependent mechanisms, SCFAs can exert their effects by regulating epigenetic processes and inflammatory transcription. Butyrate and propionate can modulate histone acetylation status, thereby altering gene-expression patterns in immune and epithelial cells [32]. Gut-microbiota-derived SCFAs have been shown to promote colonic regulatory T cell homeostasis, enhancing immune tolerance and mucosal immune balance [32]. Experimental studies showing that butyrate induces peripheral regulatory T cell (Treg cell) differentiation further indicate that SCFAs can influence forkhead box protein 3 (Foxp3)-associated immunoregulatory networks through epigenetic mechanisms [33,34].

SCFA receptors and their physiological functions are provided in Table 2.

### 2.3. SCFAs in Intestinal Barrier Function, Immune Homeostasis, and Antimicrobial Defense

At the intestinal barrier level, SCFAs act through complementary metabolic and signaling mechanisms. Butyrate serves as an important energy substrate for colonocytes and promotes tight-junction assembly, while SCFA-dependent AMPK and HIF-related signaling contributes to epithelial metabolic adaptation and barrier integrity [27,28]. SCFAs can also promote mucus-associated defense and interact with GPR43 and GPR109A-dependent pathways that regulate epithelial and mucosal homeostasis [35,36,37]. Together, these processes help restrict the translocation of luminal microorganisms and microbial products across the intestinal epithelium.

With respect to immune homeostasis, SCFA signaling is not uniformly anti-inflammatory but is dependent on receptor expression, cell type, concentration, and local inflammatory conditions. SCFAs can support Treg differentiation and mucosal immune tolerance through receptor-dependent and HDAC-related mechanisms [32,33,34,35,36], while GPR41/GPR43 activation in epithelial and innate immune cells can also regulate chemokine production and leukocyte recruitment [38]. Thus, SCFAs participate in the calibration, rather than simple suppression, of mucosal immune responses.

SCFAs are also linked to antimicrobial defense. They can regulate neutrophil migration and inflammatory mediator production [39,40], whereas microbiota-dependent signals contribute to neutrophil homeostasis, hematopoiesis, and systemic antibacterial responses [41,42,43]. In addition, microbiota-driven IgG and IgA responses contribute to protection against systemic and polymicrobial infection [44,45,46,47]. These findings suggest that SCFA-producing microbial ecosystems can influence antimicrobial defense through both direct metabolite signaling and broader microbiota-dependent immune mechanisms.

### 2.4. Mechanisms Underlying Reduced SCFA Production in Sepsis

Sepsis can reduce intestinal SCFA production through several interacting mechanisms. Gut dysbiosis, characterized by loss of microbial diversity, depletion of obligate anaerobic commensals, and expansion of opportunistic pathogens, directly reduces SCFA-producing capacity [46,47,48,49]. In addition, impaired intestinal perfusion and motility, fasting or insufficient enteral nutrition, and reduced availability of fermentable substrates may further impair microbial SCFA production.

Antibiotic exposure represents another important contributor. Broad-spectrum antibiotics, particularly those with anti-anaerobic activity, can deplete SCFA-producing commensals and promote pathogen-dominated microbial communities [50,51]. However, concern about these microbiome effects should not compromise appropriate antimicrobial treatment. In life-threatening sepsis, initial antibiotic selection should prioritize adequate coverage according to the suspected infection source, pathogens, disease severity, and resistance risk. Once microbiological and clinical information becomes available, de-escalation or narrowing of antimicrobial coverage should be considered when clinically appropriate to minimize unnecessary microbiome disruption.

Thus, preservation of SCFA-producing bacteria should complement rather than compete with effective infection control. SCFA- or microbiome-restorative strategies may be considered as adjunctive approaches once appropriate antimicrobial therapy has been initiated, with the goal of reducing preventable microbiome injury and restoring microbial metabolic function.

### 2.5. Pathological Significance of Reduced SCFA Levels

SCFA depletion is not only a consequence of gut dysbiosis but may also contribute to the progression of sepsis. Reduced butyrate availability can compromise epithelial energy metabolism and tight-junction integrity, thereby increasing intestinal permeability and facilitating the translocation of lipopolysaccharide (LPS), bacterial components, and other gut-derived inflammatory signals [26,27,28]. Reduced SCFA availability may also impair Treg-associated immune regulation [32,33,34], alter neutrophil homeostasis and function [39,40,41,42,43], and weaken microbiota-dependent mucosal and systemic antibody responses [44,45].

## 3. Mechanistic Roles of SCFAs in the Regulation of Sepsis

SCFAs exert effects in sepsis that are not limited to local metabolic alterations within the gut, but instead extend across a continuous pathological process involving gut dysbiosis, barrier disruption, uncontrolled immune-inflammatory responses, metabolic reprogramming, and organ injury. Multiple clinical and animal studies have shown that sepsis is associated with reduced gut microbial diversity, depletion of SCFA-producing bacteria such as *Roseburia*, *Bifidobacterium*, *Faecalibacterium*, *Coprococcus*, *Blautia*, *Clostridium*, *Ruminococcus*, and *Anaerostipes*, and relative expansion of opportunistic pathogens including *Enterobacteriaceae*, *Enterococcus*, and *Klebsiella*. This ecological imbalance is often accompanied by decreased levels of acetate, propionate, and butyrate, and is associated with disease severity and adverse outcomes [17,25,52]. Therefore, alterations in SCFA concentrations may provide a metabolic indication of impaired gut microbial function and may contribute to the dysregulated host response during sepsis. However, the extent to which individual SCFAs act as causal regulators rather than biomarkers of broader microbiome disruption remains to be fully established.

### 3.1. Role of SCFAs in Supporting Intestinal Barrier Integrity

Intestinal barrier disruption is a key step through which the gut shifts from a “defensive organ” to an “inflammatory amplifier” during sepsis. SCFAs can provide energy for colonocytes, promote epithelial cell proliferation, differentiation, and repair, and maintain tight-junction protein expression, mucus-layer integrity, and antimicrobial peptide production, thereby reducing the entry of bacteria, LPS, and other microbe-associated molecular patterns into the circulation [26,53,54]. In sepsis models, GPR43 deficiency aggravates intestinal barrier injury, increases pro-inflammatory cytokine levels, and reduces survival, whereas microbiota restoration or supplementation with related metabolites can partially reverse these alterations, suggesting that the SCFA–GPR43 axis is an important component in maintaining intestinal homeostasis [55,56,57].

The protective effects of SCFAs on the intestinal barrier are also reflected in their regulation of tight junction proteins and mucosal immunity. Multiple studies have shown that increasing SCFA levels or restoring SCFA-producing microbial communities upregulates tight junction proteins such as zonula occludens-1 (ZO-1), occludin, and claudin-1, reduces markers of intestinal barrier injury including diamine oxidase (DAO), D-lactate, and zonulin, and improves mucin production, secretory IgA, and local immune cell function [58,59]. In addition, infection-induced IL-1β signaling can reshape intestinal ecological niches and promote the depletion of SCFA-producing bacteria, while antibiotic-associated microbiota disruption can markedly reduce butyrate-producing bacteria such as clostridial taxa, further weakening colonization resistance and barrier defense [60,61]. In neonatal and specific infection models, restoration of gut commensal structure and SCFA production is likewise associated with alleviation of intestinal inflammation, improved microbial homeostasis, and enhanced antimicrobial defense, suggesting that this mechanism has broad relevance across different ages and disease contexts [62,63,64].

### 3.2. Context-Dependent Immunomodulation and Immunometabolic Reprogramming by SCFAs

Immune dysregulation in sepsis is characterized by dynamic and overlapping alterations in inflammatory signaling, antimicrobial defense, immune-cell phenotype, and cellular metabolism. Therefore, the effects of SCFAs should not be interpreted simply as suppression of inflammation, but rather as context-dependent modulation of host immune responses. During the early phase of sepsis, excessive activation of innate immune pathways, including toll-like receptor 4 (TLR4)/nuclear factor kappa-B (NF-κB), mitogen-activated protein kinase (MAPK), and NOD-like receptor family pyrin domain-containing 3 (NLRP3) inflammasome signaling, promotes the production of inflammatory mediators such as tumor necrosis factor-alpha (TNF-α), interleukin-1 beta (IL-1β), interleukin-6 (IL-6), and interleukin-18 (IL-18). Although acetate, propionate, and butyrate are collectively classified as SCFAs, they differ in their receptor preferences and intracellular mechanisms. Acetate predominantly signals through FFAR2/GPR43, whereas propionate can activate both FFAR2/GPR43 and FFAR3/GPR41. Butyrate also participates in receptor-mediated signaling, including GPR109A-related pathways, but exerts prominent intracellular effects through HDAC inhibition and epigenetic regulation. These distinct signaling properties may contribute to differences in their immunomodulatory effects. For example, butyrate promotes alternatively activated (M2) macrophage polarization and inhibits NF-κB and NLRP3 inflammasome activation, whereas acetate can attenuate inflammation-associated injury in aged sepsis through the FFAR2/NLRP3 axis [16,65,66].

At the molecular level, acetate, propionate, and butyrate have been associated with changes in inflammation-related targets such as NF-κB, TLR4, inducible nitric oxide synthase (iNOS), and nicotinamide adenine dinucleotide phosphate (NADPH) oxidase, together with reduced inflammatory mediator production and oxidative stress [67,68,69]. Consistent with these mechanisms, experimental interventions that improve the gut microbial environment and increase SCFA availability are frequently accompanied by reduced pro-inflammatory cytokine production, inhibition of TLR4/NF-κB signaling, attenuation of NLRP3 inflammasome activation, and improvement of histopathological injury in sepsis models [70,71,72]. SCFAs may also contribute to the regulation of epithelial and systemic immune responses by suppressing the NF-κB/myosin light chain kinase (MLCK)/myosin light chain (MLC) pathway and by modulating immune imbalance during later stages of sepsis [73,74,75]. These observations suggest that the immunological effects of SCFAs extend beyond a single anti-inflammatory pathway and instead involve coordinated regulation of inflammatory signaling and tissue–immune interactions.

It should be noted that the immunomodulatory effects associated with SCFAs may vary according to their concentrations and the stage of sepsis. Short-term exposure to relatively high concentrations of acetate can suppress hypoxia-inducible factor 1α (HIF-1α)-dependent glycolysis in macrophages and reduce pro-inflammatory mediator production, whereas under other experimental conditions acetate has been reported to aggravate pulmonary epithelial injury by altering neutrophil apoptosis and endoplasmic reticulum stress [76,77]. This context dependence is closely linked to immunometabolic reprogramming. For example, GPR43 deficiency can promote a pro-inflammatory classically activated (M1) macrophage phenotype accompanied by increased HIF-1α and enolase 1 (ENO1) expression, whereas SCFA-related signaling can suppress this glycolytic program and partially reverse the pro-inflammatory phenotype [55]. Thus, SCFAs can influence inflammatory responses not only by acting on canonical signaling pathways but also by modifying the metabolic programs that determine immune-cell function.

The consequences of SCFA signaling also differ among innate immune-cell populations. Acetate can enhance neutrophil chemotaxis, oxidative burst, phagocytic receptor expression, and bacterial clearance, whereas butyrate can increase the proportion of CX3C chemokine receptor 1-positive (CX3CR1^+^) natural killer (NK) cells and promote interferon-gamma (IFN-γ) production, thereby supporting antimicrobial defense [78,79]. SCFAs also participate in adaptive immune regulation. Sodium butyrate has been shown to increase CD4^+^Foxp3^+^ regulatory T (Treg) cells while improving intestinal and pulmonary barrier function and attenuating lung injury in cecal ligation and puncture (CLP) models [21]. In clinical nutrition and immune-reconstitution studies, SCFA levels have been associated with immunoglobulin concentrations, T-cell subsets, and nutritional status, suggesting a potential relationship between SCFA availability and immune recovery in critically ill patients [80,81,82]. Single-cell and multi-omics analyses further indicate that propionate-related metabolic states are associated with plasma-cell transcriptional profiles, B-cell–neutrophil interactions, transcription-factor activity, and sepsis-related diagnostic signatures, whereas SCFA-related genes are enriched primarily in monocytes and are associated with immune infiltration and glycerophospholipid metabolic alterations [83,84,85].

Taken together, these findings suggest that SCFAs function as context-dependent immunometabolic regulators that can influence inflammatory signaling, antimicrobial effector functions, immune-cell differentiation, and cellular energy metabolism. Their net effects are therefore likely to depend on the specific SCFA, its local concentration, receptor expression, immune-cell phenotype, tissue environment, and the temporal stage of sepsis.

### 3.3. Epigenetic Regulation, Post-Translational Protein Modifications, and Mitochondrial Homeostasis

SCFAs can couple microbial substrate availability to cellular transcriptional and metabolic programs through epigenetic and post-translational mechanisms. Butyrate and propionate can alter chromatin accessibility by inhibiting HDAC activity and increasing histone acetylation [32], thereby modifying the transcription of genes involved in inflammation, epithelial barrier function, immune-cell differentiation, and cell survival. In parallel, changes in intracellular acetyl-CoA availability can influence the acetylation of histone and non-histone proteins, including regulatory proteins involved in NF-κB signaling. Thus, SCFA-dependent protein acetylation provides a mechanism through which microbial metabolism can be translated into changes in host-cell phenotype.

These epigenetic and post-translational effects are closely linked to mitochondrial homeostasis. Butyrate can serve as a metabolic substrate and has been reported to preserve mitochondrial respiratory capacity and electron-transport-chain function in several experimental settings [86,87]. Improved mitochondrial redox balance may subsequently reduce lipid peroxidation and ferroptosis-related injury, whereas GPR109A-related signaling can influence transcription factor EB (TFEB)-dependent autophagic flux [88,89,90]. However, these mitochondrial effects are not uniformly beneficial across experimental settings. Notably, Peters et al. [91] demonstrated that intravenous butyrate supplementation exacerbated mitochondrial dysfunction in both cardiomyocytes and immune cells in a rat model of fecal peritonitis. This negative finding contrasts with the protective effects observed in LPS-based models and indicates that the biological consequences of butyrate are highly dependent on the disease model and treatment context. In particular, differences in the route of administration, systemic exposure, dose, timing of intervention, stage and severity of sepsis, and host metabolic status may substantially influence whether SCFA supplementation is beneficial or detrimental. Therefore, SCFAs should not be regarded as universally protective metabolites, and beneficial effects observed with one route, dose, or disease model should not be directly extrapolated to other clinical settings. From a translational perspective, this discrepancy highlights the need for systematic pharmacokinetic and dose–response studies, route-specific safety evaluation, and identification of stage-specific therapeutic windows before systemic SCFA supplementation can be considered for clinical application in sepsis. Thus, the impact of SCFAs on mitochondrial function depends on metabolic context, tissue, dose, and route of administration.

### 3.4. Acting Together with Other Microbial Metabolites to Form a Host-Protective Network

**Figure 1:**
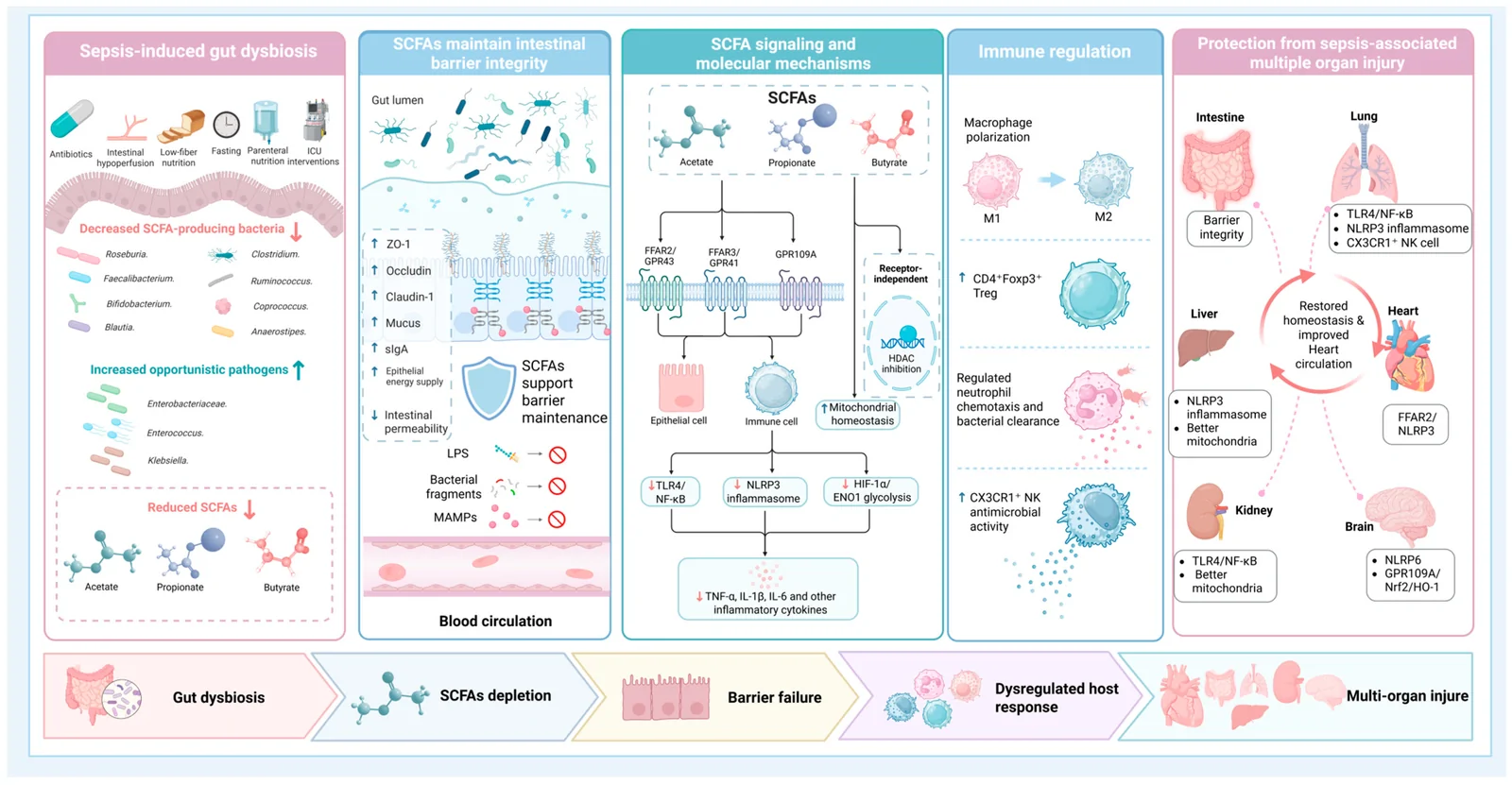
SCFAs regulate the gut microbiota–barrier–immunometabolism–organ axis during sepsis. Sepsis-associated gut dysbiosis and reduced SCFA production may impair intestinal barrier integrity and promote systemic inflammation. SCFAs may modulate immune and metabolic responses through SCFA receptors and downstream signaling pathways, thereby influencing sepsis-associated multiple-organ injury.

Although this review focuses on SCFAs, the metabolic effects of the gut microbiota in sepsis often result from the coordinated actions of multiple metabolites. Tryptophan metabolites, such as indole-3-propionic acid (IPA) and indole-3-acetic acid (IAA), can influence macrophage phagocytosis, T cell function, liver injury, myocardial inflammation, and neuroinflammation through pathways involving aryl hydrocarbon receptor (AhR), pregnane X receptor (PXR), and NF-κB/NLRP3, indirectly highlighting the systemic regulatory value of gut-derived metabolites in sepsis [92,93,94]. In models of lung injury, bacteremia, and sepsis-associated encephalopathy (SAE), metabolites such as IPA or IAA exert protective effects by modulating gut microbiota composition, enhancing immune cell function, or suppressing microglial inflammatory responses [95,96,97]. Evidence from these adjacent microbial metabolites helps place SCFAs within the broader “gut microbiota–metabolite–host immunity” network, rather than interpreting their effects in isolation [98,99] (Figure 1).

## 4. SCFAs and Sepsis-Associated Multiple Organ Injury

The development of sepsis-associated multiple-organ injury is closely linked to systemic inflammation, endotoxin translocation, endothelial injury, microcirculatory dysfunction, immunometabolic dysregulation, and mitochondrial dysfunction. The unique feature of SCFAs is that, although they are derived from the gut microbiota, they can affect distant organs through the portal vein, lymphatic circulation, and peripheral blood, thereby serving as important metabolic links in organ axes such as the gut–lung, gut–heart, gut–liver, gut–kidney, and gut–brain axes.

### 4.1. Intestinal Injury

The gut is not only the primary site of SCFA production and utilization, but also one of the earliest organs injured during sepsis and a persistent amplifier of systemic inflammation. Sepsis can induce abnormal intestinal villus architecture, crypt alterations, reduced microbial diversity, and decreased fecal SCFA levels, changes that often coexist with cognitive impairment, elevated inflammatory status, and adverse outcomes [100,101]. In the context of increased susceptibility to bloodstream infection, depletion of butyrate-producing bacteria, reduced small-intestinal SCFA levels, and abnormal tight junction protein expression collectively facilitate bacterial entry into the bloodstream, whereas SCFA supplementation can ameliorate this process [26]. These findings indicate that SCFA deficiency is not only a consequence of sepsis-associated gut dysbiosis, but also an important contributor to intestinal barrier failure.

### 4.2. Lung Injury

The gut–lung axis represents an important pathway potentially linking SCFAs to sepsis-associated lung injury. In sepsis-associated acute lung injury, higher SCFA availability has been associated with reduced gut-derived LPS translocation, lower activity of TLR4/NF-κB and NLRP3 signaling, altered neutrophil and macrophage responses, and improved alveolar epithelial barrier function [21,69,89]. Butyrate can enhance the migration and antimicrobial activity of CX3CR1^+^ NK cells, thereby helping control *Klebsiella pneumoniae*-associated lung injury and bacterial burden [79]. Consistently, GPR43 signaling has been shown to contribute to pulmonary host defense against *Klebsiella pneumoniae* infection, further supporting the involvement of SCFA-sensing pathways in antibacterial immunity in the lung [102].

Antibiotic-associated microbiota disruption can increase opportunistic pathogens and antibiotic resistance genes while reducing levels of acetate, propionate, butyrate, and other beneficial fatty acids, thereby impairing the reparative capacity of the alveolar epithelial barrier. Restoration of microbial metabolic function can ameliorate pulmonary inflammation and tissue injury [103]. In addition, infection can disturb gut microbiota composition and metabolic function, indicating that the systemic consequences of pulmonary infection are closely linked to alterations in the intestinal ecosystem [104]. Some studies also suggest that modulation of microbiota-related metabolites can improve sepsis-associated lung injury, and that these effects are associated with changes in SCFA-producing bacteria, bile acids, or other lipid metabolites [92,95,105].

### 4.3. Myocardial Injury

Septic cardiomyopathy involves excessive release of inflammatory cytokines, mitochondrial dysfunction, disordered energy metabolism, calcium homeostasis abnormalities, and cardiomyocyte death. SCFAs may contribute to myocardial protection by suppressing inflammation, maintaining intestinal barrier integrity, improving metabolic status, and modulating modes of cell death. In aged animal models of sepsis, low acetate levels are associated with microbiota fragility, organ dysfunction, and poor prognosis, whereas increasing acetate can attenuate myocardial inflammation and functional impairment through the FFAR2/NLRP3 axis [66]. Butyrate supplementation can reduce oxidative stress and inhibit ferroptosis, thereby ameliorating sepsis-induced myocardial injury [88].

SCFAs may also influence cardiac rhythm stability during sepsis. Sodium acetate and sodium butyrate can prevent bacterial outer membrane vesicle-induced sepsis-associated arrhythmias through FFAR2/FFAR3-related signaling, suggesting that the cardioprotective effects of SCFAs may extend beyond anti-inflammatory actions and metabolic support to include maintenance of electrophysiological homeostasis [106]. However, under specific administration conditions, butyrate may also aggravate mitochondrial dysfunction in cardiomyocytes and immune cells. Therefore, its myocardial protective potential requires further validation in relation to the experimental model, dose, and route of administration [91].

### 4.4. Hepatic and Renal Injury

The liver is directly exposed to gut-derived metabolites through the portal circulation, making the gut–liver axis particularly relevant during sepsis. SCFAs and related microbial metabolites may influence hepatic inflammation, mitochondrial function, and innate immune responses. However, compared with its effects on renal mitochondrial respiration, butyrate appears to have a relatively limited effect on hepatic mitochondrial respiration [87].

The kidney is also highly susceptible to systemic inflammation, endotoxemia, and metabolic disturbance during sepsis. Experimental enhancement of intestinal SCFA production can strengthen intestinal barrier function, reduce systemic LPS exposure, and attenuate sepsis-associated acute kidney injury through inhibition of TLR4/NF-κB signaling [107]. Increased SCFA availability has also been associated with reduced renal inflammatory cytokine production and modulation of vitamin D-related pathways [108]. Moreover, butyrate can preserve renal mitochondrial respiratory capacity and electron-transport-chain function following inflammatory stress [87], suggesting that renal protection may involve both immunological and metabolic mechanisms.

### 4.5. Brain Injury

Sepsis-associated encephalopathy is closely linked to gut dysbiosis, blood–brain barrier dysfunction, microglial activation, and neuroinflammation. SCFAs may regulate peripheral inflammation and central immune responses through the gut–brain axis, thereby influencing delirium, anxiety-like behavior, and cognitive dysfunction. Studies have shown that sepsis can disrupt intestinal barrier integrity and NLRP6 inflammasome function, thereby aggravating hippocampal neuroinflammation, whereas SCFAs can attenuate these alterations through an NLRP6-dependent mechanism [109]. In SAE models of different severities, higher butyrate production is associated with milder neuroinflammation, and sodium butyrate can reduce microglial oxidative stress and improve survival through the GPR109A/nuclear factor erythroid 2-related factor 2 (Nrf2)/heme oxygenase-1 (HO-1) pathway [110]. In addition, modulation of SCFA-producing bacteria can ameliorate sepsis-associated neurological injury.

### 4.6. Muscle, Endothelial, and Systemic Metabolic Injury

Sepsis not only damages major parenchymal organs, but also causes muscle weakness, endothelial barrier dysfunction, and systemic metabolic disturbances. Butyrate can improve muscle-related pathological processes by repairing the intestinal mucosa, suggesting that restoration of the gut barrier may indirectly influence distant tissue function [111]. Endothelial barrier injury and immunothrombosis are also important components of sepsis progression, and related lipid metabolites can influence neutrophil and platelet adhesion, suggesting that future studies should further explore the relationship between SCFAs, endothelial homeostasis, and coagulation-associated inflammation [112].

## 5. Therapeutic Advances in SCFA-Related Interventions

The primary goal of SCFA-related therapeutic strategies is not merely to supplement a single metabolite, but to restore the functional “gut microbiota–SCFAs–host immunometabolism” axis. Current studies mainly focus on direct SCFA supplementation, promotion of endogenous SCFA production, restoration of SCFA-producing microbial communities, targeting of SCFA receptors, and the use of SCFAs as indicators for risk stratification. Given the marked heterogeneity of sepsis, future therapeutic approaches should emphasize patient stratification, timing of intervention, and safety assessment.

### 5.1. Direct Supplementation with SCFAs and Related Metabolites

Direct supplementation with acetate, butyrate, or mixed SCFAs represents one of the most extensively investigated SCFA-related intervention strategies. In direct experimental studies, acetate administration has been associated with enhanced neutrophil antibacterial activity through GPR43 and with improved outcomes in severe *Staphylococcus* aureus infection models. Short-term exposure to relatively high concentrations of acetate has also been linked to reduced pro-inflammatory responses and alterations in HIF-1α-dependent glycolysis in macrophages [76,78]. In experimental sepsis in aged animals, acetate supplementation has been associated with changes in gut microbial composition, reduced organ dysfunction, and attenuation of myocardial injury, together with modulation of the FFAR2/NLRP3 axis [66]. These findings suggest that acetate may warrant further investigation in settings characterized by impaired acetate availability or age-related microbial dysbiosis, although the relevance of these observations to specific patient populations remains to be established.

Evidence regarding butyrate and sodium butyrate has primarily focused on epithelial barrier function, inflammatory regulation, and organ injury. In experimental studies, butyrate administration has been associated with reduced myocardial ferroptosis-related changes, improved pulmonary barrier parameters, and neuroprotective phenotypes involving the GPR109A/Nrf2/HO-1 pathway [21,88,110]. Sodium acetate and sodium butyrate have also been associated with reduced occurrence of sepsis-associated arrhythmias in experimental models [90,106]. Importantly, these beneficial associations are not universal. Intravenous butyrate administration aggravated mitochondrial dysfunction in cardiomyocytes and immune cells in a severe peritonitis model [91]. This contrasting result indicates that the effects of direct metabolite supplementation may vary substantially according to dose, route of administration, timing, disease stage, systemic exposure, and tissue metabolic context.

### 5.2. Promoting Endogenous SCFA Production Through Nutritional Support

Nutritional support is an important determinant of intestinal substrate availability and microbial SCFA production. In patients with sepsis, poorer tolerance of enteral feeding has been associated with lower SCFA concentrations, supporting a relationship between intestinal nutrient delivery and microbial fermentative activity [113]. Different nutritional modalities have also been associated with distinct changes in the gut microbiota and SCFA profiles. Total enteral nutrition appears to be accompanied by greater increases in several SCFAs, whereas parenteral nutrition has shown more limited associations with improvements in SCFA concentrations and immune-related parameters [81]. In neonatal early-onset sepsis, early alterations in SCFA profiles have also been observed, suggesting possible time-dependent relationships among nutrient availability, microbial maturation, and infection risk [114].

Furthermore, probiotic intervention studies have shown that improvements in inflammatory or endotoxemia-related parameters are not necessarily accompanied by substantial changes in circulating SCFA concentrations [82]. This dissociation indicates that the effects of nutritional and microbiome-targeted interventions should not be attributed solely to increases in plasma SCFAs. Future studies should therefore assess SCFA concentrations in feces, plasma, and relevant tissues together with microbial functional profiles, nutritional exposure, and host immune parameters.

### 5.3. Restoring SCFA-Producing Microbial Communities and Microecological Function

Restoration of SCFA-producing microbial communities represents an indirect strategy for modifying SCFA availability and intestinal microbial function. Taxa including *Bifidobacterium*, *Lactobacillus*, *Faecalibacterium*, *Blautia*, *Lachnospiraceae*, and *Clostridia* have been associated with SCFA production, intestinal barrier-related functions, and inflammatory regulation [58,115,116]. In several experimental studies, restoration or enrichment of these microbial communities, together with stabilization of or increases in fecal butyrate and propionate levels, has been associated with reduced intestinal injury, lower inflammatory activity, and less severe sepsis-related phenotypes [75,117,118]. Because these interventions simultaneously alter microbial composition and multiple metabolic products, however, these findings do not establish SCFAs as the sole mediators of the observed effects.

FMT and other forms of microbial reconstruction can alter microbial diversity and SCFA-related metabolic capacity. In sepsis models involving antibiotic-induced dysbiosis, FMT and direct SCFA supplementation were each associated with reduced mortality, improved inflammatory parameters, and changes in pyroptosis-related signaling [16]. In *Klebsiella pneumoniae* pneumonia-induced sepsis, microbiota restoration has been associated with higher levels of acetate, propionate, butyrate, and secondary bile acids, together with reduced alveolar barrier injury and systemic inflammation [103]. Similarly, in experimental sepsis in aged animals, transplantation of microbiota from young donors has been associated with increased *Akkermansia* abundance and acetate levels as well as improved outcomes [66]. These observations suggest that the metabolic characteristics of the donor microbiota may influence the biological effects of microbiome-based interventions, although acetate itself cannot be assumed to account for the entire therapeutic association.

Evidence from additional microbial-transfer studies also suggests that protective phenotypes can be associated with transferable microbial or metabolite profiles [59,99]. Despite encouraging findings in experimental models, FMT should currently be regarded as an investigational rather than routine intervention in sepsis. Critically ill and immunocompromised patients may be particularly vulnerable to transmission of infectious or antimicrobial-resistant organisms and to unpredictable ecological effects after introduction of complex donor-derived microbial communities. Moreover, ongoing broad-spectrum antibiotic therapy may interfere with donor microbiota engraftment. Therefore, the clinical use of FMT in sepsis requires rigorous donor screening, careful patient selection, standardized protocols, and prospective safety evaluation before any therapeutic recommendation can be made.

### 5.4. Targeting SCFA Receptors and Downstream Signaling Pathways

Compared with direct SCFA supplementation, targeting SCFA receptors and downstream signaling pathways may offer greater therapeutic control. FFAR2/GPR43 plays an important role in maintaining intestinal barrier integrity, regulating macrophage glycolysis, inhibiting NLRP3 inflammasome activation, and ameliorating myocardial injury [55,66]. FFAR2 can also be upregulated by vitamin D receptor (VDR), thereby reducing lipid peroxidation in macrophages and attenuating lung injury, suggesting that modulation of receptor expression may represent a novel immunometabolic target [89]. GPR109A is closely associated with the antioxidant, autophagy-enhancing, and neuroprotective effects induced by butyrate and 3-hydroxybutyrate [90,110].

**Figure 2:**
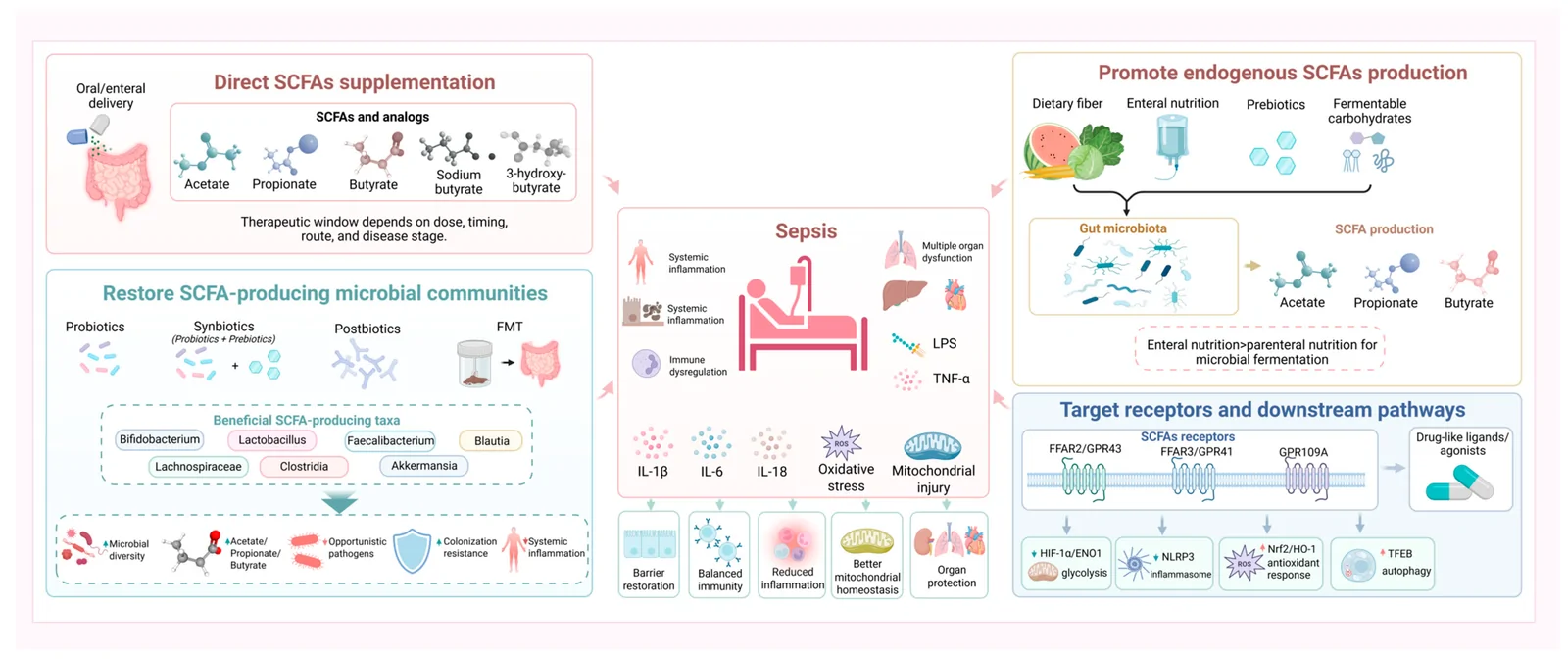
Therapeutic strategies targeting the SCFA–microbiota–immunometabolism axis in sepsis. Potential approaches include direct SCFA supplementation, promotion of endogenous SCFA production, restoration of SCFA-producing microbial communities, and targeting of SCFA receptors and downstream signaling pathways.

Beyond receptors, HDACs, NF-κB, NLRP3, HIF-1α, ENO1, MLCK, TFEB, and ferroptosis-related molecules may together constitute the downstream intervention network of SCFAs. Acetate regulates macrophage inflammatory responses through acetyl-CoA generation and HIF-1α-dependent glycolysis, whereas butyrate modulates inflammasome activation, antioxidant responses, and autophagy through GPR41 or GPR109A. These provide candidate molecules for future targeted therapy. Propionate metabolism is closely associated with plasma cell transcriptional states and immune cell interactions [65,76,83]. Multi-omics analyses have also identified SCFA-associated molecular signatures involving genes such as *CASP5*, *GPR84*, and *MMP9* [84,85]. At present, these genes should be regarded primarily as biomarkers or hypothesis-generating candidates rather than validated downstream effectors or therapeutic targets of SCFAs. It remains unclear whether their expression is directly regulated by acetate, propionate, or butyrate, whether they mediate SCFA-dependent biological effects, or merely reflect broader inflammatory and metabolic changes during sepsis. Functional studies using targeted genetic or pharmacological manipulation will therefore be required to establish causality and determine whether any of these molecules can be therapeutically exploited within the SCFA–host response axis (Figure 2).

### 5.5. SCFAs as Biomarkers and a Basis for Precision Therapy

SCFAs and their associated microbial communities have potential value as indicators for sepsis risk stratification, prognostic assessment, and therapeutic monitoring. In ICU patients with sepsis, enteral nutrition tolerance, microbial characteristics, and SCFA levels are correlated, indicating that SCFAs may be used to assess intestinal function and responses to nutritional therapy [113]. In non-ICU patients with bacteremia-associated sepsis, reductions in protective microbial taxa and decreased acetate and propionate levels further support the combined use of SCFAs and microbiota-based indicators for infection risk assessment [17].

From a dynamic prognostic perspective, patients with sepsis exhibit persistent changes in gut microbiota and metabolites across different stages of ICU treatment. Low butyrate levels, high *Klebsiella* abundance, elevated taurocholic acid, and higher SOFA scores together indicate an increased risk of mortality [25]. Studies in Zhuang patients with sepsis and in neonatal sepsis have both suggested that SCFA-related microbial communities and metabolic profiles show identifiable population-specific signatures, which may enable early recognition and individualized stratification when combined with machine-learning models in the future [52,114]. Within the framework of precision medicine, reduced microbial diversity, expansion of opportunistic pathogens, decreased SCFA production, barrier injury, and high inflammatory burden may together define a patient phenotype that is particularly suitable for SCFA-related interventions [119].

The primary source of infection should also be considered when evaluating SCFA-related abnormalities and therapeutic responses. Abdominal, pulmonary, urinary, and bloodstream infections differ in their degree of intestinal involvement, pathogen spectrum, antimicrobial requirements, and host immune responses. Therefore, alterations in SCFA production and the response to SCFA-related interventions may not be equivalent across different sources of sepsis. Future clinical studies should stratify patients according to infection source rather than treating sepsis as a biologically uniform condition.

Overall, SCFA-related therapy has conceptually evolved from “supplementation with a single metabolite” to an integrated strategy aimed at reconstructing gut microbiota function, restoring barrier homeostasis, modulating immunometabolism, and protecting multiple organ functions. Future studies should use prospective cohorts, dynamic metabolic monitoring, and mechanism-oriented clinical trials to clarify the functional boundaries of different SCFAs across distinct stages of sepsis, and to establish standardized systems for dose, route of administration, efficacy endpoints, and safety evaluation.

### 5.6. Clinical Translational Challenges and Future Directions

Despite promising preclinical findings, several practical barriers limit the clinical translation of SCFA-based therapies in sepsis. First, enteral administration may be unreliable in critically ill patients because gastrointestinal dysmotility, ileus, intestinal hypoperfusion, and feeding intolerance can affect delivery and absorption. Although impaired enteral nutrition tolerance has been associated with lower SCFA levels in patients with sepsis [113], the pharmacokinetics and bioavailability of enterally administered SCFAs in this population remain poorly defined.

Second, parenteral administration also raises important safety concerns. No validated therapeutic concentration or safety window has been established for intravenous SCFAs in sepsis, and intravenous butyrate has been reported to aggravate mitochondrial dysfunction in a fecal peritonitis model [91]. These findings indicate that the effects of SCFAs may depend strongly on dose, route of administration, systemic exposure, and disease stage.

Third, restoration of endogenous SCFA production is complicated by the continued use of broad-spectrum antibiotics, particularly agents with anti-anaerobic activity, which can deplete SCFA-producing commensals [50,51]. Because appropriate antimicrobial treatment remains essential in sepsis, microbiome-directed strategies should complement rather than replace antibiotic therapy. Potential approaches include antimicrobial de-escalation when clinically appropriate, restoration of fermentable substrates, and microbiota-independent strategies such as direct SCFA or postbiotic supplementation.

Finally, treatment responses are unlikely to be uniform across all patients with sepsis. Infection source, pathogen, immune phenotype, organ dysfunction, antibiotic exposure, baseline microbiota, and endogenous SCFA levels may all influence therapeutic effects. However, direct evidence comparing SCFA responses among defined sepsis subtypes remains limited. Future clinical studies should therefore incorporate patient stratification and biomarker-guided selection to identify individuals most likely to benefit from SCFA-related interventions.

## 6. Conclusions

SCFAs are important molecular links between the gut microbiota and host immunometabolism, exerting multilayered regulatory effects during the development and progression of sepsis. During sepsis, intestinal hypoperfusion, inflammatory injury, antibiotic exposure, insufficient nutritional substrates, and ICU-related interventions can reduce SCFA-producing bacteria and decrease metabolites such as acetate, propionate, and butyrate. SCFA deficiency further compromises intestinal epithelial energy supply and tight junction integrity and promotes increased intestinal permeability, LPS translocation, and amplification of systemic inflammation, thereby contributing to the development of multiple organ dysfunction.

Mechanistically, SCFAs can mediate signal transduction through receptors such as FFAR2/GPR43, FFAR3/GPR41, and GPR109A, and can also influence gene expression and cellular function through epigenetic regulation. Their effects involve the maintenance of intestinal barrier integrity, regulation of Treg differentiation, modulation of neutrophil and macrophage function, improvement of mitochondrial injury, and control of processes such as inflammasome activation, autophagy, pyroptosis, and ferroptosis. Through these mechanisms, SCFAs are closely associated with sepsis-related lung injury, myocardial injury, hepatic and renal injury, intestinal injury, and sepsis-associated encephalopathy.

Therapeutically, direct SCFA supplementation, promotion of endogenous SCFA production, restoration of SCFA-producing microbial communities, and modulation of SCFA receptors and their downstream pathways have all shown application prospects. Butyrate and sodium butyrate are currently the most extensively studied intervention forms, while acetate, propionate, dietary fiber, probiotics, synbiotics, postbiotics, and FMT also provide new directions for adjunctive therapy in sepsis. However, current evidence is still mainly derived from basic research and small-sample studies, and clinical efficacy, safety, optimal dose, timing of administration, and suitable patient populations remain to be clarified.

Overall, current evidence supports an association between SCFA-related metabolism and several components of the dysregulated host response in sepsis, but it does not yet establish SCFA supplementation or microbiome-directed restoration as effective clinical therapy. The effects of SCFAs are likely to depend on infection source, host immune and metabolic status, gastrointestinal function, antibiotic exposure, route and timing of intervention, and the origin and disposition of circulating SCFAs. Prospective, stratified clinical studies are therefore required before SCFA-based interventions can be recommended for routine clinical use.

## Figures and Tables

**Table 1:** Major Short-Chain Fatty Acids: Microbial Sources, Metabolic Characteristics, Physiological and Immunological Features, and Alterations in Sepsis.

SCFA Type	Major Sources/Producing Bacteria	Major Metabolic Fate	Major Physiological/Immunological Features	Alterations in Sepsis	References
Acetate	Various anaerobic bacteria, including *Bifidobacterium* spp.	The most abundant SCFA in the gut; after intestinal absorption, acetate enters the portal circulation and can subsequently reach the systemic circulation	Associated with the regulation of neutrophil chemotaxis, oxidative burst, phagocytic activity, and inflammatory responses	Often decreased in the context of gut dysbiosis; reduced acetate levels have been reported in patients with sepsis	[17,22,25]
Propionate	*Bacteroides* spp. and certain Bacillota	After intestinal absorption, propionate enters the portal circulation and undergoes substantial hepatic metabolism	Associated with immune regulation, metabolic homeostasis, and epigenetic regulation, including HDAC-related effects	Reduced propionate levels have been reported in patients with sepsis and critical illness	[17,22,25]
Butyrate	*Faecalibacterium*, *Roseburia*, *Clostridium*, *Anaerostipes*, and related taxa	Extensively oxidized and utilized as an energy substrate by colonocytes, resulting in relatively limited entry into the systemic circulation	Closely associated with epithelial barrier integrity, histone acetylation, and the induction and maintenance of Foxp3^+^ regulatory T cells	Butyrate-producing bacteria and fecal butyrate levels are often reduced during sepsis-associated gut dysbiosis	[17,25,26]

**Table 2:** SCFA Receptors and Their Physiological Functions.

SCFA Receptor/Pathway	Major Ligands	Physiological Functions	Mechanisms of Action	References
FFAR2/GPR43	Acetate, propionate, butyrate.	Regulates inflammatory responses and neutrophil function.	Mediates SCFA sensing and controls immune-cell chemotaxis and inflammation resolution.	[29]
FFAR3/GPR41	Propionate, butyrate.	Regulates energy metabolism and neuroendocrine signaling.	Modulates sympathetic nervous system activity and host energy homeostasis.	[29,31]
GPR109A	Butyrate, niacin.	Suppresses intestinal inflammation and maintains mucosal immune tolerance.	Promotes anti-inflammatory signaling and intestinal homeostasis.	[35,36]
Histone Deacetylase (HDAC) Inhibition Pathway	Butyrate, propionate.	Promotes immune tolerance and regulatory T-cell differentiation.	Inhibits histone deacetylases and enhances Foxp3^+^ Treg generation.	[33]
AMPK/Hypoxia-inducible factor (HIF)-Related Pathway	Butyrate.	Maintains epithelial barrier integrity.	Enhances tight-junction assembly and promotes epithelial hypoxia adaptation.	[28]

## Data Availability

No new data were created or analyzed in this study. Data sharing is not applicable to this article.
